# 19S Proteasome Subunits as Oncogenes and Prognostic Biomarkers in FLT3-Mutated Acute Myeloid Leukemia (AML)

**DOI:** 10.3390/ijms232314586

**Published:** 2022-11-23

**Authors:** Joshua J. Lara, Alfonso E. Bencomo-Alvarez, Mayra A. Gonzalez, Idaly M. Olivas, James E. Young, Jose L. Lopez, Vanessa V. Velazquez, Steven Glovier, Mehrshad Keivan, Andres J. Rubio, Sara K. Dang, Jonathan P. Solecki, Jesse C. Allen, Desiree N. Tapia, Boranai Tychhon, Gonzalo E. Astudillo, Connor Jordan, Darshan S. Chandrashekar, Anna M. Eiring

**Affiliations:** 1 Paul L. Foster School of Medicine, Texas Tech University Health Sciences Center at El Paso, El Paso, TX 79905, USA; 2 L. Frederick Francis Graduate School of Biomedical Sciences, Texas Tech University Health Sciences Center at El Paso, El Paso, TX 79905, USA; 3 Center of Emphasis in Cancer, Department of Molecular and Translational Medicine, Texas Tech University Health Sciences Center at El Paso, El Paso, TX 79905, USA; 4 Department of Pathology-Molecular & Cellular, University of Alabama at Birmingham, Birmingham, AL 35233, USA

**Keywords:** acute myeloid leukemia (AML), FMS-like tyrosine kinase 3 (FLT3), proteasome 26S subunit non-ATPase 3 (PSMD3), proteasome inhibition

## Abstract

26S proteasome non-ATPase subunits 1 (PSMD1) and 3 (PSMD3) were recently identified as prognostic biomarkers and potential therapeutic targets in chronic myeloid leukemia (CML) and multiple solid tumors. In the present study, we analyzed the expression of 19S proteasome subunits in acute myeloid leukemia (AML) patients with mutations in the FMS-like tyrosine kinase 3 (*FLT3*) gene and assessed their impact on overall survival (OS). High levels of *PSMD3* but not *PSMD1* expression correlated with a worse OS in FLT3-mutated AML. Consistent with an oncogenic role for PSMD3 in AML, shRNA-mediated PSMD3 knockdown impaired colony formation of FLT3+ AML cell lines, which correlated with increased OS in xenograft models. While PSMD3 regulated nuclear factor-kappa B (NF-*κ*B) transcriptional activity in CML, we did not observe similar effects in FLT3+ AML cells. Rather, proteomics analyses suggested a role for PSMD3 in neutrophil degranulation and energy metabolism. Finally, we identified additional *PSMD* subunits that are upregulated in AML patients with mutated versus wild-type FLT3, which correlated with worse outcomes. These findings suggest that different components of the 19S regulatory complex of the 26S proteasome can have indications for OS and may serve as prognostic biomarkers in AML and other types of cancers.

## 1. Introduction

Acute myeloid leukemia (AML) is a heterogeneous leukemic disease characterized by abnormal stem cells in the bone marrow. AML stem cells result in the accumulation of immature myeloid cells in the peripheral blood or bone marrow, which ultimately leads to bone marrow failure [1]. Despite initial high response rates to standard induction chemotherapy regimens, most patients eventually relapse and succumb to their disease [1]. Molecular profiling of AML through cytogenetics and targeted sequencing panels has revealed the biological complexity of this disease and is used for risk stratification and guiding therapeutic options for patients [2]. For example, alterations in the FMS-like tyrosine kinase-3 (*FLT3*) gene are common driver mutations of AML and are present in over 30% of all cases. Patients with FLT3 internal tandem duplications (FLT3-ITDs) have a poor prognosis, with higher relapse rates and reduced overall survival (OS) [1]. Upon the binding of FLT3 ligand, the FLT3 receptor tyrosine kinase induces hematopoietic stem and progenitor cell mobilization in vivo, and knockout of Flt3 in mice was shown to be non-lethal [2]. Therefore, FLT3 tyrosine kinase inhibitors (TKIs) such as quizartinib, midostaurin, or gilteritinib are logical choices for treatment in FLT3+ AML [3,4]. Although FLT3 inhibitors can improve remission rates of AML patients, up to 60% experience drug toxicity or acquired resistance mechanisms, such as point mutations in the tyrosine kinase domain (TKD) that render FLT3 inhibitors ineffective [5,6,7]. Strategies to overcome resistance have been reported, such as synergistic drug combinations that incorporate FLT3 inhibitors with the standard chemotherapy drug regimen [8,9]. However, identifying novel prognostic biomarkers and therapeutic targets will be essential to countering resistance mechanisms and providing a better prognosis for AML patients [8,10].

An alternative approach to circumvent these treatment barriers and provide a potential target for combination therapies is the ubiquitin-proteasome system (UPS), a multi-protein complex that plays an essential role in regulating protein homeostasis, cell cycle progression, and apoptosis [6]. The 26S proteasome is comprised of two main subcomplexes: the 19S regulatory complex and the 20S core complex, which together recognize ubiquitylated proteins to facilitate their UPS-dependent degradation [11,12]. The 19S regulatory complex serves dual roles by (1) recognizing and binding ubiquitylated proteins and (2) unfolding and translocating those peptides into the 20S core complex for degradation [13]. Bortezomib (Velcade^®^) is a reversible inhibitor of chymotrypsin-like proteasome activity, which functions by binding to and inhibiting the 20S beta 5 subunit of the proteasome (β5, PSMB5) [14]. Bortezomib is used as standard therapy in treatment-refractory multiple myeloma by blocking the 20S core complex, leading to the accumulation of ubiquitylated proteins and ultimately cell death [15]. Notably, previous studies have shown that FLT3+ AML cells are more sensitive to bortezomib than AML cells harboring wild-type FLT3 [16,17]. Therefore, the UPS is a promising target for the development of therapeutic drugs with efficacy in FLT3+ AML. However, similar to FLT3 TKIs, proteasome inhibitors such as bortezomib are prone to acquired resistance mechanisms, possibly due to mutations in the molecular target of bortezomib, PSMB5, which further highlights the need for alternative therapeutic strategies [18].

Timely UPS-dependent signal molecule degradation is crucial in both normal and leukemic hematopoiesis and differentiation [19]. In AML, overexpression of the 20S beta 8 subunit of the proteasome (β8, *PSMB8*) was reported to predict an unfavorable prognosis [20]. We previously reported a role for two members of the 19S regulatory complex, 26S proteasome non-ATPase subunits 1 (*PSMD1*) and 3 (*PSMD3*), in disease progression and drug resistance of chronic myeloid leukemia (CML) and several types of solid tumors [11,21]. Importantly, an oncogenic role for PSMD1 and PSMD3 had already been reported in breast cancer [22,23]. In our study, *PSMD1* and *PSMD3* mRNA expression was markedly upregulated in patients who had progressed from the indolent chronic phase to the rapidly fatal blast phase of CML. Knockdown of either protein induced apoptosis of CML cell lines and patient samples, with no effect on normal cord blood controls [21], suggesting they may be good targets for therapy. Ultimately, our data demonstrated that these proteins play an oncogenic role in CML by stabilizing the nuclear factor-kappa B (NF-κB) transcription factor [21]. Similarly, we found that *PSMD1* and *PSMD3* mRNA expression is upregulated in multiple cancer types compared with normal tissue. High levels of *PSMD1* or *PSMD3* mRNA expression correlated with worse OS in various solid tumors, such as breast invasive carcinoma, colon adenocarcinoma, and kidney renal papillary cell carcinoma, to name a few [11]. Thus, we hypothesized that non-ATPase members of the 19S proteasome could serve as novel prognostic biomarkers and potential therapeutic targets in FLT3-mutated AML.

In the present study, we used data from The Cancer Genome Atlas (TCGA) available at UALCAN (http://ualcan/path.uab.edu/, accessed on 29 July 2021) and Gene Expression Profiling Interactive Analysis 2 (GEPIA2) (http://gepia2.cancer-pku.cn/, accessed on 2 August 2021) to analyze the expression of non-ATPase subunits of the 19S proteasome (Table 1) in patients with FLT3-mutated AML.

We found that the expression of *PSMD3* but not *PSMD1* correlated with worse OS in FLT3+ AML. We further assessed the potential for PSMD3 to act as an oncogene in FLT3+ AML both in vitro and in vivo, and whether other 19S proteasome subunits could serve as novel prognostic biomarkers. Collectively, our findings suggest that different components of the 19S regulatory complex in the 26S proteasome have indications for OS and may serve as prognostic biomarkers and novel therapeutic targets in FLT3-mutated AML and other types of cancers.

## 2. Results

### 2.1. High Levels of PSMD3 but Not PSMD1 mRNA Expression Correlated with Worse OS in AML Patients with Mutated versus Wild-Type FLT3

**Figure 1:**
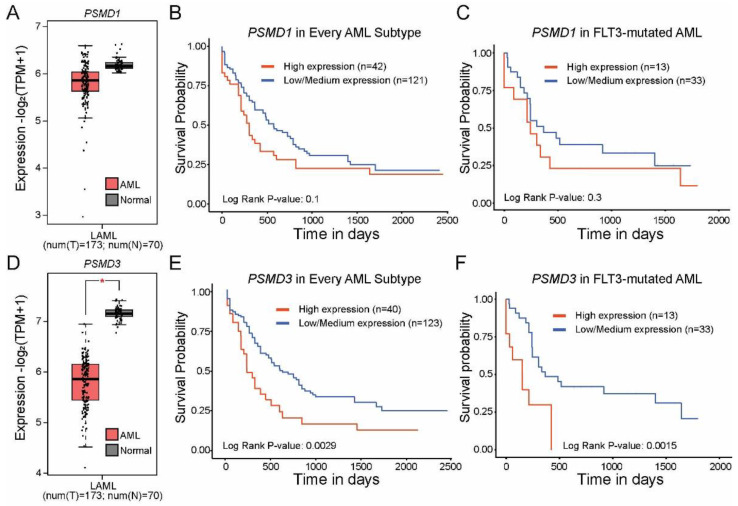
High levels of *PSMD3* but not *PSMD1* mRNA expression correlated with worse overall survival (OS) in FLT3-mutated AML. (**A**) The box plot shows *PSMD1* expression in AML versus normal mononuclear cells (MNCs) from GEPIA2. Error bars represent standard error of the mean (SEM, *p* = ns). (**B**,**C**) Kaplan–Meier curves show the effect of *PSMD1* mRNA expression on OS in AML when all subtypes are considered (**B**) and in FLT3-mutated AML (**C**) from UALCAN. (**D**) The box plot shows *PSMD3* expression in AML versus normal MNCs from GEPIA2. Error bars represent SEM (* *p* < 0.001). (**E**,**F**) Kaplan–Meier curves show the effect of *PSMD3* mRNA expression on OS in AML when all subtypes are considered (**E**) and in FLT3-mutated AML (**F**) from UALCAN.

Based on our recent data in CML and other types of cancers [11,21], we hypothesized that *PSMD1* and *PSMD3* mRNA expression would be upregulated in AML versus normal mononuclear cells (MNCs), and that high expression would correlate with a worse OS. In fact, Dai et al. previously reported that high *PSMD3* mRNA levels were associated with worse outcomes in AML [24]. Using data from GEPIA2, we observed no significant difference in *PSMD1* expression comparing AML versus normal mononuclear cells (Figure 1A). Consistent with this report, data from UALCAN revealed no correlation in OS comparing AML patients with high versus low *PSMD1* expression (*p* = 0.1, Figure 1B). Taking these data one step further, we used UALCAN to analyze OS between FLT3-mutated AML patients with high versus low *PSMD1* expression, which again had no effect on OS (*p* = 0.3, Figure 1C). Data from GEPIA2 surprisingly showed that *PSMD3* mRNA expression levels were significantly downregulated in AML versus normal controls (*p* < 0.001, Figure 1D). Despite this observation, TCGA data available at UALCAN showed that high levels of *PSMD3* mRNA expression correlated with a significant reduction of OS when all AML subtypes were considered (*p* = 0.0029, Figure 1E), and this was especially true for AML patients with FLT3 mutations (*p* = 0.0015, Figure 1F). Altogether, these data implicate PSMD3 as a potential oncogene in FLT3-mutated AML.

### 2.2. PSMD3 Knockdown Reduced Survival of FLT3-Mutated AML Cell Lines In Vitro and In Vivo

**Figure 2:**
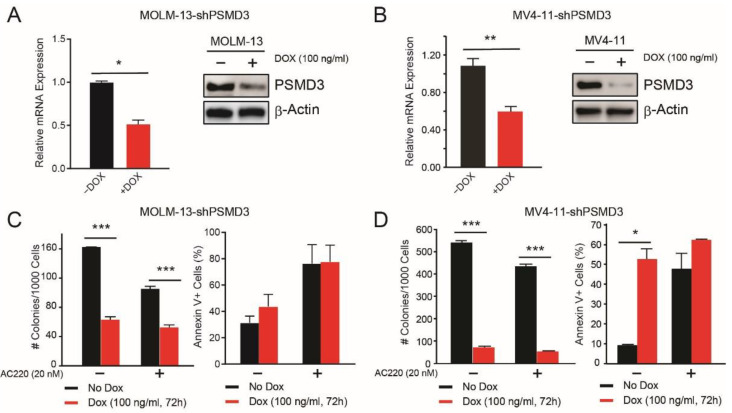
shRNA-mediated PSMD3 (shPSMD3) knockdown impaired survival of the FLT3-mutated AML cell lines, MOLM-13 and MV4-11, with variable effects on apoptosis. (**A**,**B**) Bar graphs and immunoblots show PSMD3 knockdown at the mRNA and protein level, respectively, in MOLM-13 (**A**) and MV4-11 (**B**) cells cultured with and without doxycycline (DOX, 100 ng/mL, 72 h). (**C**,**D**) Bar graphs show the effect of shPSMD3 on survival in colony formation (**left**) and late apoptosis (**right**) in MOLM-13 (**C**) and MV4-11 (**D**) cells cultured ± doxycycline (DOX, 100 ng/mL, 72 h) and ± quizartinib (AC220, 20 nM, 72 h). Error bars represent SEM (* *p* < 0.05; ** *p* < 0.01; *** *p* < 0.001).

We have already reported that shRNA-mediated knockdown of PSMD3 had no effect on survival or apoptosis of normal cord blood CD34^+^ progenitor cells, but reduced survival and increased apoptosis of CML cell lines in vitro and in vivo [21]. To assess the role of PSMD3 in AML, we transduced the FLT3-mutated AML cell lines, MOLM-13, MOLM-14, and MV4-11 with doxycycline-inducible shPSMD3, and confirmed PSMD3 knockdown at the mRNA and protein level in the presence of doxycycline (Figure 2A,B, Supplementary Figure S1A). Consistent with an oncogenic role for PSMD3 in FLT3-mutated AML, shRNA-mediated knockdown of PSMD3 impaired survival of all three cell lines in colony formation assays in vitro, in the presence and absence of the FLT3 TKI, quizartinib (also known as AC220, Figure 2C,D,left, Supplementary Figure S1B, left). However, while shPSMD3 increased apoptosis of CML cell lines in the presence and absence of therapy [21], the phenotype was different in FLT3-mutated AML cell lines. Rather, in the absence of the FLT3 TKI, quizartinib, shPSMD3 significantly increased apoptosis of MOLM-14 and MV4-11 cells, but not MOLM-13 cells, with no further effects when cells were cultured with TKIs (Figure 2C,D,right, Supplementary Figure S1B, right).

**Figure 3:**
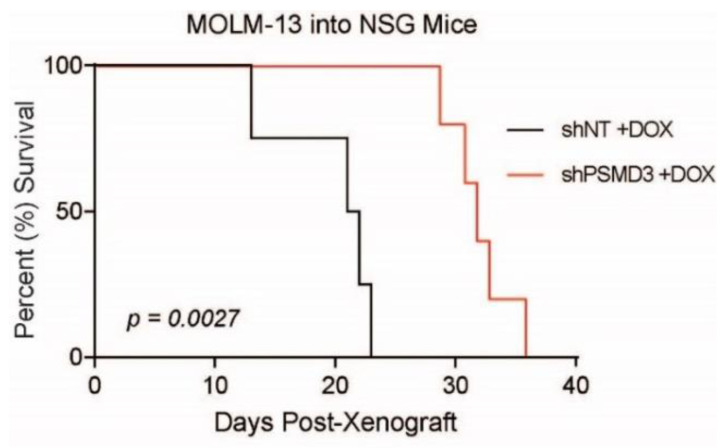
shPSMD3 enhanced survival of MOLM-13 cells engrafted into NOD-scid IL2Rgamma^null^ (NSG) recipient mice. Kaplan–Meier curve shows overall survival (OS) of sublethally irradiated NSG recipient mice upon intravenous injection of MOLM-13 cells (3 × 10^6^) expressing either shPSMD3 (*n* = 5) or the non-targeting shNT control vector (*n* = 4) and treated with doxycycline (DOX, 625 mg/kg).

To confirm these data using an in vivo model, we intravenously injected 3 × 10^6^ shNT- or shPSMD3-expressing MOLM-13 cells into sublethally irradiated NOD-scid IL2Rgamma^null^ (NSG) recipient mice and assessed for differences in OS in the presence of doxycycline. Consistent with an oncogenic role for PSMD3 in AML, mice receiving shPSMD3-expressing cells had a significant increase in OS compared with mice receiving the shNT-expressing control cells (*p* = 0.0027, Figure 3). Altogether, these data suggest an oncogenic role for PSMD3 in promoting survival of FLT3-mutated AML cells both in vitro and in vivo. However, the effect of PSMD3 knockdown on apoptosis was variable, with significant effects in the MOLM-14 and MV4-11 cell lines, but no effect in MOLM-13 cells, implying alternative oncogenic functions compared with our previous observations in CML [21].

### 2.3. PSMD3 Knockdown in FLT3+ AML Resulted in An Increase of Global Ubiquitylated Proteins, but Had Little Effect on NF-κB Luciferase Reporter Activity

**Figure 4:**
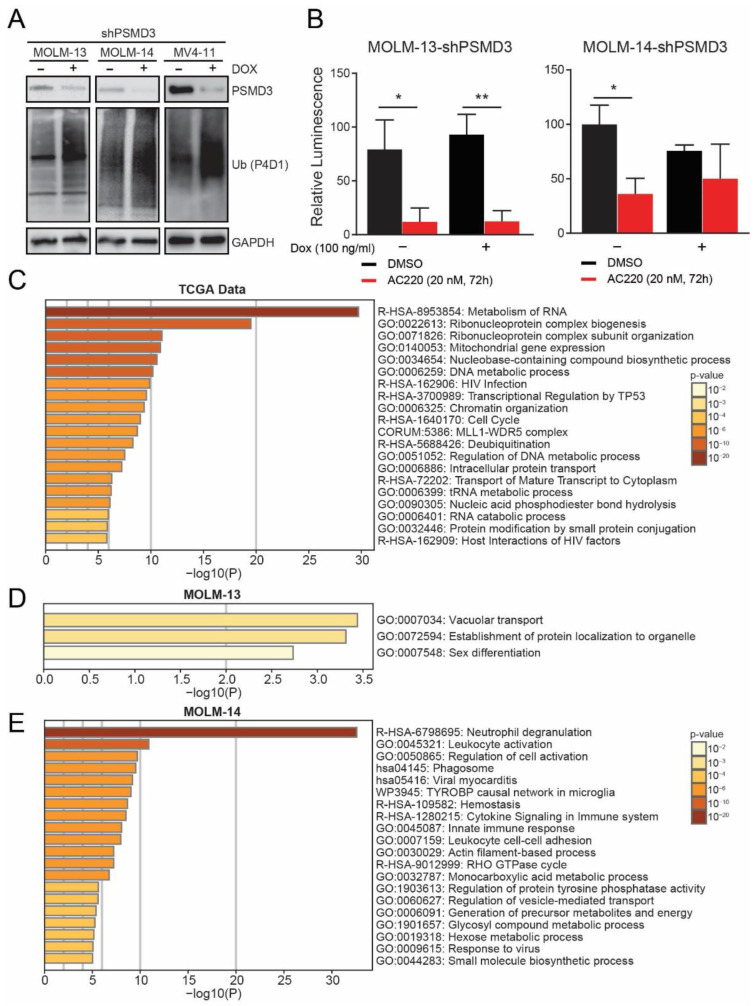
PSMD3 knockdown in FLT3+ AML increased global ubiquitylated proteins, but had little effect on NF-κB luciferase reporter activity. (**A**) Immunoblot shows the effect of PSMD3 knockdown (shPSMD3) on global protein ubiquitylation in MOLM-13, MOLM-14, and MV4-11 cells. GAPDH levels were measured as a control. (**B**) Bar graphs show the effect of PSMD3 knockdown combined with the FLT3 TKI, quizartinib (AC220, 20 nM, 72 h), on NF-κB luciferase reporter activity in MOLM-13 (**left**) and MOLM-14 (**right**) cells. Error bars represent SEM (* *p* < 0.05; ** *p* < 0.01). (**C**) Bar graph shows pathway enrichment analysis of the gene sets upregulated with *PSMD3* expression in AML data from The Cancer Genome Atlas (TCGA) available at UALCAN (http://ualcan/path.uab.edu/, accessed on 30 July 2021). (**D**,**E**) Bar graphs show pathway enrichment analysis of the proteins that were dysregulated upon shRNA-mediated PSMD3 knockdown in the MOLM-13 (**D**) and MOLM-14 (**E**) AML cell lines, as measured by mass spectrometry-based proteomics analyses. The darkness of the orange color reflects the *p*-value, with darker colors having greater significance.

In CML, we previously reported that PSMD1 or PSMD3 knockdown decreased NF-κB luciferase reporter activity, which correlated with a global accumulation of ubiquitylated proteins [21]. Similarly, knockdown of PSMD3 in FLT3+ AML cells correlated with an increased level of global ubiquitylated proteins (Figure 4A). Therefore, we speculated that knockdown of PSMD3 would decrease NF-κB transcriptional activity in FLT3+ AML cells. Surprisingly, luciferase reporter assays demonstrated that, while quizartinib showed the expected reduction of NF-κB luciferase reporter activity, PSMD3 knockdown had no significant effect (Figure 4B). However, in MOLM-14 cells, shPSMD3 showed a trend for reduced NF-κB transcriptional activity, which was not further reduced by quizartinib therapy (Figure 4B; *p* = 0.07). These data further suggest that the role of PSMD3 in FLT3+ AML cells is distinct from its function in BCR-ABL1+ CML cells.

To address the oncogenic role of PSMD3 in FTL3+ AML, we used the correlation feature available at UALCAN to calculate the gene sets upregulated with *PSMD3* in TCGA AML data (Supplementary Figure S2). Our analysis implicated a potential role for PSMD3 in the following pathways: metabolism of RNA (R-HSA-8953854), ribonucleoprotein complex biogenesis (GO:0022613), ribonucleoprotein complex subunit organization (GO:0071826), mitochondrial gene expression (GO:0140053), nucleobase-containing compound biosynthetic process (GO:0034654), and DNA metabolic process (GO:0006259), among others (Figure 4C). To corroborate these results at the protein level, we performed mass spectrometry-based proteomics analyses in MOLM-13 and MOLM-14 cells after PSMD3 knockdown. In the MOLM-14 AML cell line, PSMD3 knockdown resulted in a significant increase of 149 proteins and a decrease of 265 proteins. In contrast, PSMD3 knockdown in the MOLM-13 cell line resulted in an increase of only 25 proteins and a decrease of only 8 proteins. The leading proteins that were up- or down-regulated upon PSMD3 knockdown in both cell lines are listed in Supplementary Tables S1 and S2. Interestingly, when pathway enrichment analysis was performed on the upregulated proteins, the leading dysregulated pathways were vacuolar transport (G0:0007034) in the MOLM-13 cell line and neutrophil degranulation (R-HSA-6798695) in the MOLM-14 cell line (Figure 4D,E). In proteomics analyses, additional pathways dysregulated by shPSMD3 included several metabolic pathways, such as the monocarboxylic acid metabolic process, generation of precursor metabolites and energy, glycosyl compound metabolic process, hexose metabolic process, and small molecule biosynthetic process (Figure 4E).

### 2.4. Expression of PSMD2, PSMD6, PSMD7, and PSMD9 Are Elevated in AML Patients with Mutated versus Wild-Type FLT3, Which Correlated with Worse Outcomes

**Figure 5:**
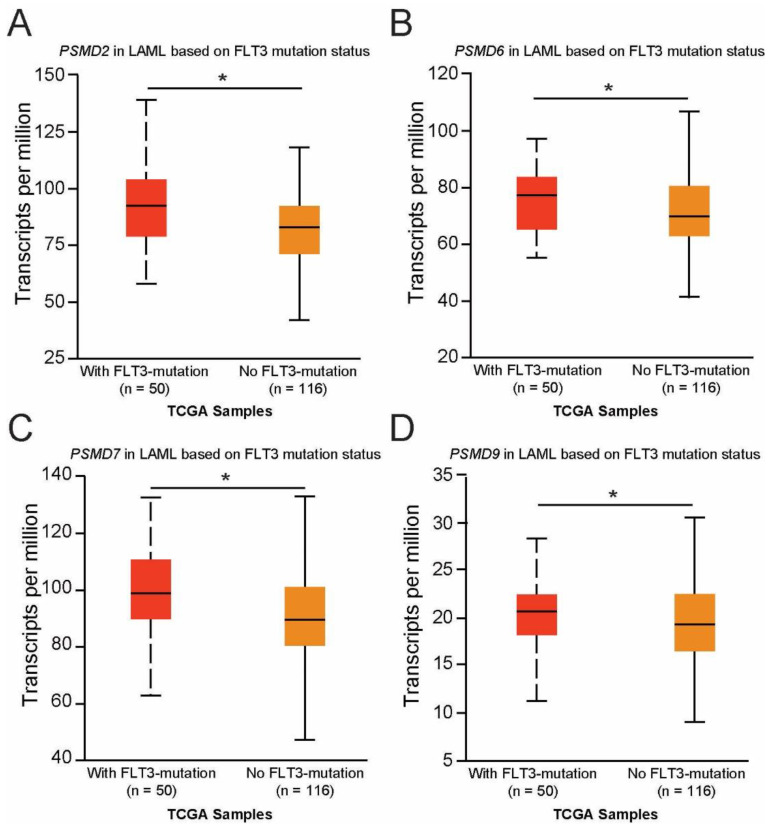
Correlation of *PSMD2*, *PSMD6*, *PSMD7*, and *PSMD9* mRNA expression with FLT3 mutation status in AML. (**A**–**D**) We used TCGA data available at UALCAN to associate the expression of other *PSMD* subunits with OS in AML. The box plots demonstrate that mRNA encoding PSMD2 (**A**), PSMD6 (**B**), PSMD7 (**C**), and PSMD9 (**D**) were significantly elevated in AML patients with mutated versus wild-type FLT3. Error bars represent SEM (* *p* < 0.05).

We next asked whether the expression of other non-ATPase subunits of the 19S proteasome were altered in AML patients with mutated versus wild-type FLT3 and whether they correlate with OS. TCGA data available from UALCAN demonstrated that the genes encoding PSMD2 (*p* = 0.046), PSMD6 (*p* = 0.03), PSMD7 (*p* = 0.046), and PSMD9 (*p* = 0.048) were found to be significantly upregulated in AML patients with mutated versus wild-type FLT3 (Figure 5A–D).

**Figure 6:**
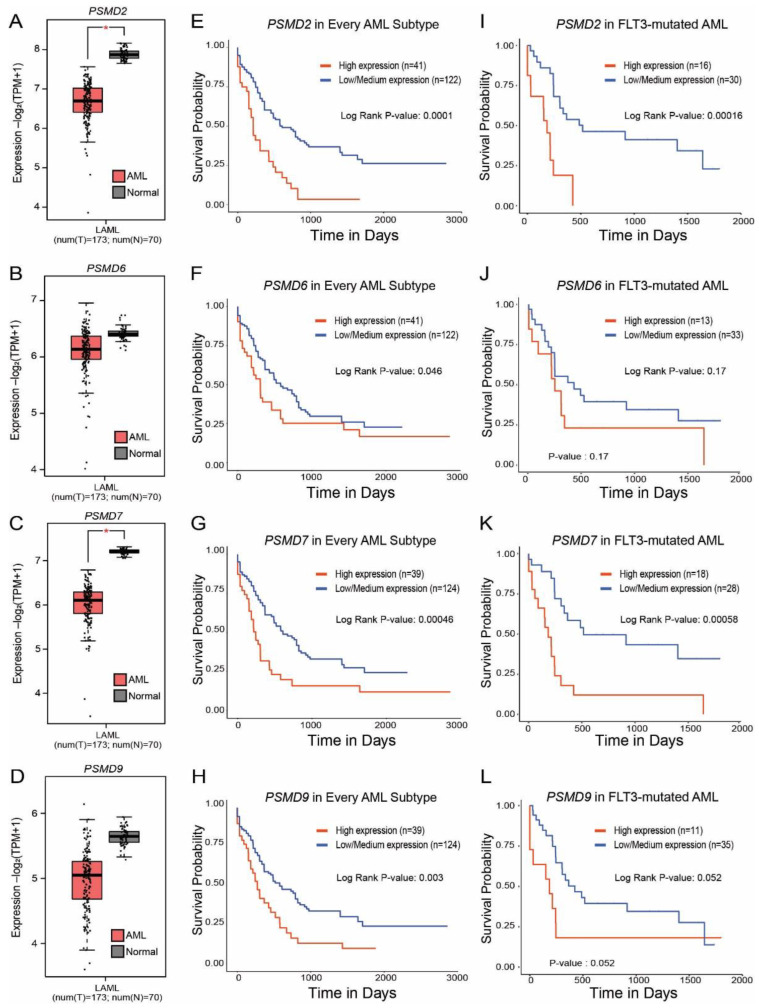
High levels of *PSMD2* and *PSMD7* mRNA expression correlated with worse outcomes in FLT3-mutated AML. (**A**–**D**) Box plots show *PSMD2* (**A**), *PSMD6* (**B**), *PSMD7* (**C**), and *PSMD9* (**D**) mRNA expression in AML versus normal MNCs. (**E**–**H**) Kaplan–Meier curves show OS for AML patients with high versus low levels of *PSMD2* (**E**), *PSMD6* (**F**), *PSMD7* (**G**), and *PSMD9* (**H**) when all AML subtypes are considered. (**I**–**L**) Kaplan–Meier curves show OS for FLT3-mutated AML patients with high versus low levels of *PSMD2* (**I**), *PSMD6* (**J**), *PSMD7* (**K**), and *PSMD9* (**L**). Error bars represent SEM (* *p* < 0.001).

Similar to *PSMD3* (Figure 1C), GEPIA2 mRNA expression data showed that *PSMD2* and *PSMD7* (*p* < 0.01), but not *PSMD6* or *PSMD9*, were surprisingly downregulated in AML versus normal MNCs (Figure 6A–D). When all AML subtypes were considered, high expression of mRNA encoding PSMD2 (*p* = 0.0001), PSMD6 (*p* = 0.046), PSMD7 (*p* = 0.00046), and PSMD9 (*p* = 0.003) correlated with a worse OS in TCGA AML data (Figure 6E–H). In FLT3-mutated AML, high levels of *PSMD2* (*p* = 0.00016) and *PSMD7* (*p* = 0.00058), but not *PSMD6* (*p* = 0.17) or *PSMD9* (*p* = 0.052) mRNA expression correlated with a significant reduction in OS (Figure 6I–L). Additionally, mRNA encoding other non-ATPase UPS subunits, including PSMD4 (*p* = 0.044), PSMD8 (*p* = 0.0032), and PSMD13 (*p* = 0.00022) also correlated with reduced OS in AML (Supplemental Figure S3A–C). Altogether, while expression of many *PSMD* subunits is reduced in AML versus normal MNCs, AML patients with elevated expression of several of these 19S subunits correlates with poor outcomes.

Thus far, our data suggest an oncogenic role for 19S PSMD subunits in FLT3-mutated AML. Using cBioPortal to analyze multiple TCGA cancers, we previously reported that altered *PSMD1* expression was associated with mutations or deep deletions in several different solid tumors, whereas *PSMD3* was primarily associated with gene amplifications [11]. Therefore, we hypothesized that genomic alterations could be responsible for the differences in expression of these 19S subunits in AML. In contrast, for the non-ATPase subunits of the 19S proteasome that were reported here to correlate with OS in AML (*PSMD2*, *PSMD3*, *PSMD4*, *PSMD6*, *PSMD7*, *PSMD8*, *PSMD9*, and *PSMD13*; Figure 1, Figure 6 and Figure S3), cBioPortal revealed very few reported genomic alterations. The only genes with reported genomic alterations were deep deletions in *PSMD7* and *PSMD13* (Figure S4). Otherwise, the remaining 19S proteasome subunit genes had no genomic alterations reported in AML, suggesting alternative methods for regulation of these genes in FLT3+ AML.

## 3. Discussion

AML is a complex, heterogeneous disease that initially responds well to standard chemotherapy drug regimens, yet despite initial responses, patients often relapse due to the presence of common driver mutations. Mutations in the FLT3 tyrosine kinase receptor are common in AML, and using FLT3 TKIs has shown promising results for many AML patients [25,26,27,28]. Unfortunately, most patients eventually develop resistance to FLT3 inhibitors, making FLT3 a poor prognostic biomarker in this disease. One novel strategy to circumvent this method of resistance is to selectively target the UPS. The UPS is a vital cellular organelle involved in the regulation of protein homeostasis [20]. In AML cells with FLT3 mutations, proteasome inhibitors such as bortezomib are more effective compared with cells harboring wild-type FLT3, but are still prone to resistance mechanisms [16,17]. Recent work by Mo et al. has shown that knockdown of the proteasome subunit, PSMB5, improved bortezomib sensitivity in patients with multiple myeloma through the increased expression of the pro-apoptotic signaling gene, BAX, and decreased expression of the anti-apoptotic genes, BCL2 and AKT [29].

Another potential strategy to overcome drug resistance in cancer therapy is to target the 19S regulatory complex instead of the 20S core complex (such as PSMB5), which is the target of standard proteasome inhibitors such as bortezomib [14]. For example, several groups have demonstrated the potential for targeting adhesion-regulating molecule 1 (ADRM1/hRPN13) in cancer therapy and bortezomib resistance [30,31,32,33,34,35,36]. This rigorous work has led to the development of the putative RPN13 inhibitor, RA190 [37,38,39,40]. However, more work must be done before we can effectively target the 19S regulatory particle as a therapeutic approach in AML.

We have previously reported that the proteasome subunits, *PSMD1* and *PSMD3*, are upregulated during progression of CML from the indolent chronic phase to the aggressive blast phase of the disease [21]. We also previously showed that *PSMD1* and *PSMD3* mRNA expression are upregulated in other types of cancers compared with normal tissue, and that this often correlates with a worse prognosis [11]. In the present study, we assessed whether *PSMD1*, *PSMD3*, and other non-ATPase subunits of the 19S proteasome could serve as prognostic biomarkers and potential therapeutic targets in FLT3+ AML. Using publicly available databases, we analyzed *PSMD1* and *PSMD3* expression in FLT3-mutated AML patients and found no significant difference in OS comparing patients with high versus low *PSMD1* expression (Figure 1C). However, while *PSMD3* mRNA levels were significantly reduced in AML versus normal cells, patients with higher *PSMD3* expression levels demonstrated a significant reduction in OS, especially for AML patients harboring mutated FLT3 (*p* = 0.0015) (Figure 1F). These data suggest that *PSMD3* could be a novel prognostic biomarker for risk stratification and therapy response prediction in AML. Therefore, we used shRNAs to assess the oncogenic potential of PSMD3 in FLT3+ AML cell lines in vitro and in vivo. Knockdown of PSMD3 resulted in reduced survival of FLT3+ AML cells in colony formation assays (Figure 2C,D,right), which correlated with better OS when injected into immunocompromised recipient mice (Figure 3). These findings suggest that targeting specific proteasomal subunits could be a potential therapeutic option for patients with AML and other types of cancers.

Indeed, the proteasomal subunit, *PSMD2*, has prognostic significance in patients with bladder urothelial carcinoma [41] and lung adenocarcinoma [42,43]. Other reports demonstrated a role for PSMD6 in lung adenocarcinoma [20] and esophageal squamous cell carcinoma [43]. PSMD7, a core component of the 26S proteasome, may play a role in the progression of breast cancer and could also serve as a prognostic indicator or therapeutic target [44]. *PSMD7* mRNA expression was found to be upregulated in breast cancer tissue, and knockdown led to cell cycle arrest and apoptosis by decreasing the expression of cell cycle proteins and increasing the stability of the p21 and p27 cell cycle regulators [44]. PSMD7 may also play a role in the prognosis of patients with head and neck squamous cell carcinoma. Patients with high *PSMD7* expression demonstrated poor clinical outcomes in patients with lung cancer, which also contributed to cisplatin resistance in patients with gastric cancer [45,46,47]. Additionally, recent work by Ud Din Farooqee et al. has implicated PSMD9 as a potential drug target for cancer therapy or ribosome-associated disorders [48]. PSMD9 was shown to affect two critical aspects in patients with breast cancer: (1) trafficking of ribosomal subunits into the nucleus and (2) contributing to decreased breast cancer cell growth and death. These findings implicate a structural role for PSMD9 during ribosome interactions [48]. When we analyzed whether the expression of other *PSMD* subunits also correlated with OS in AML, patients with elevated levels of *PSMD2*, *PSMD7*, and possibly *PSMD9* had worse outcomes (Figure 6I–L). High expression of *PSMD4*, *PSMD8*, and *PSMD13* also correlated with reduced OS in AML (Figure S3A–C).

In CML, we observed reduced NF-κB luciferase reporter activity with PSMD3 knockdown [21]. When we assessed the effects of PSMD3 knockdown on NF-κB luciferase activity in FLT3+ AML, we observed minimal changes (Figure 4B), suggesting that additional signaling pathways are regulated by PSMD3 expression in this particular disease. The divergent phenotypes elicited by PSMD3 knockdown in CML versus AML suggest distinct functional roles for PSMD3 in these myeloid malignancies. Indeed, proteomics analyses revealed vacuolar transport and neutrophil degranulation as the top dysregulated pathways upon knockdown of PSMD3 in FLT3+ AML cells (Figure 4D,E). This is not entirely surprising, as the PSMD3 gene on chromosome 17 is upstream of the gene encoding colony-stimulating factor 3 (CSF3), a member of the interleukin (IL)-6 superfamily of cytokines. CSF3 controls the production, differentiation, and function of human granulocytes, and mice lacking CSF3 are neutropenic [49]. In humans, common variations in the *PSMD3-CSF3* gene were previously associated with neutrophil counts [50]. Additional pathways dysregulated by PSMD3 knockdown in FLT3+ AML included immune cell cytokine signaling and energy metabolism pathways. Future studies will assess the mechanism by which these proteins contribute to survival in FLT3-mutated AML patients and whether they serve as novel targets for therapy.

Many studies have documented the prognostic significance of *PSMD* subunit expression in multiple cancers [11,20,21,22,23,44,46,48,51]. Despite their recent progress as prognostic biomarkers, more research is needed before we can effectively target the 19S proteasome as a therapeutic option in cancer. Importantly, since the PSMD subunits of the 19S proteasome are non-ATPase proteins that lack catalytically active target sites, they could be challenging molecular targets that require the blockage of protein-protein interactions. A different approach could be to target the proteasome 26S subunit, ATPase (PSMC) members of the 19S proteasome, which are ATPases that harbor catalytically active target sites. Indeed, *PSMC2*, *PSMC3*, *PSMC4*, *PSMC5*, and *PSMC6* were recently reported as highly expressed in breast cancer, which correlated with worse outcomes [52]. PSMC2 has been implicated as an oncogene in multiple cancer types, including glioma, gastric cancer, squamous cell carcinoma, and skin cutaneous melanoma, to name a few [53,54,55,56]. In colorectal cancer, PSMC5 promoted cancer cell proliferation and metastasis by activating the epithelial-mesenchymal transition and modulating immune cell infiltration [57]. A CRISPR genome-wide screen identified dependence on PSMC6 for bortezomib sensitivity in multiple myeloma [58]. Whether the PSMC and PSMD subunits of the 19S proteasome are viable targets for therapeutic intervention will be an important topic of future studies. Altogether, our data point to subunits of the 19S proteasome as novel prognostic biomarkers and putative targets for therapy in FLT3+ AML, CML, and other types of cancers that are worthy of future investigation.

## 4. Materials and Methods

### 4.1. Analysis of 19S Regulatory Subunit Expression in AML Patients Using Publicly Available Databases

GEPIA2 (http://gepia2.cancer-pku.cn/, accessed on 2 August 2021) integrates data from TCGA and the Genotype-Tissue Expression (GTEx) project to provide large-scale gene expression profiling [12,13,14]. Using GEPIA2, the differential mRNA expression of the 19S proteasomal subunits, *PSMD1* and *PSMD3*, were analyzed in AML versus normal tissue. We also performed gene expression analyses for the other non-ATPase subunits of the 19S proteasome in AML (Table 1). UALCAN (http://ualcan/path.uab.edu/, accessed on 29 July 2021) is another publicly available platform comparing transcriptome or proteomics data from either TCGA or the Clinical Proteomic Tumor Analysis Consortium (CPTAC), respectively [59]. We used UALCAN to compare PSMD subunit expression in AML patients based on clinicopathological features (mutant versus wild-type FLT3). UALCAN was also used to assess the effects of PSMD subunit expression on OS in AML patients.

### 4.2. Cell Lines

The FLT3-ITD+ AML cell lines, MOLM-13 and MOLM-14, were purchased from Deutsche Sammlung von Mikroorganismen and Zellkulteren GmbH (The Leibniz Institute DSMZ, Braunschweig, Germany). Both cell lines evolved from myelodysplastic syndrome (MDS) and carry the Casitas B-lineage lymphoma (CBL) deltaExon8 mutant and FLT3-ITD. The FLT3-ITD+ MV4-11 cell line was purchased from American Type Culture Collection (ATCC, Manassas, VA, USA). All cell lines were cultured in RPMI medium (Life Technologies Corporation, Carlsbad, CA, USA) with 10% fetal bovine serum (FBS, Life Technologies Corporation), 2.0 mM L-glutamine (Life Technologies Corporation), and 100 U/mL penicillin/streptomycin (Life Technologies Corporation). Where specified, the indicated concentrations of quizartinib (AC220, Selleck Chemicals, Houston, TX, USA) were used for FLT3 inhibition in all cell lines.

### 4.3. Lentiviral shRNA Constructs

Using vector sequences from our previously published shRNA library screen, lentiviral small hairpin RNA (shRNA) vectors targeting *PSMD3* (RefSeq: NM_002809.2) and a non-targeting control vector (shNT) were purchased from Cellecta (Mountain View, CA, USA) to incorporate as constructs for lentiviral production [21,60]. The pGreenFire1-NF-κB lentivector was purchased from System Biosciences, LLC (Palo Alto, CA, USA) (Supplemental Table S3). Lentivirus-producing 293FT cells (Life Technologies) were maintained in Dulbecco’s Modified Eagle Medium (DMEM) supplemented with 10% FBS, 2.0 mM L-glutamine, 100 U/mL penicillin/streptomycin, 1.0 mM sodium pyruvate (Life Technologies), and 0.1 mM Minimum Essential Medium (MEM) non-essential amino acids (Life Technologies Corporation). Constructs were packaged using psPAX2 and VSV.G (Supplemental Table S3). Viral particles were concentrated after binding to Polyethylene Glycol 8000 (1800 rpm, 45 min., 4 °C, Fisher Scientific, Hampton, NH, USA) and delivered to target cells by spinoculation [61]. Infected cells were selected by either fluorescence-activated cell sorting (FACS) for green fluorescent protein (GFP)-positive cells or by treatment with 2 μg/mL puromycin dihydrochloride (Thermo Fisher Scientific, Waltham, MA, USA) or Geneticin™ Selective Antibiotic (G418 Sulfate) (Life Technologies Corporation). To induce knockdown of doxycycline-inducible constructs, cells were treated ± doxycycline hyclate (100 ng/mL, 72 h, Gold Biotechnology Inc., St. Louis, MO, USA).

### 4.4. Analysis of PSMD3 mRNA Expression Using Reverse Transcription-Quantitative Polymerase Chain Reaction (RT-qPCR)

The PureLink RNA Mini Kit by Invitrogen (Thermo Fisher Scientific) was used to extract total RNA from cell lines and then quantified using a NanoDrop^TM^ 2000 (Thermo Fisher Scientific). RT-qPCR was performed using the Luna Universal One-Step qPCR Kit (New England Biolabs, Ipswich, MA, USA) on a StepOnePlus Real-Time PCR System (Applied Biosystems, Foster City, CA, USA). *GUSB* mRNA levels were measured as controls, and the primer sequences are listed in Supplemental Table S4. Each RT-qPCR assay was performed in triplicate and analyzed using the comparative cycle threshold method (2^−ΔΔCt^) to calculate the relative gene expression level of *PSMD3*.

### 4.5. Immunoblot to Confirm Knockdown of PSMD3 at the Protein Level

MOLM-13, MOLM-14, and MV4-11 cells and their derivative lines were cultured following the protocol indicated above, and the resulting cells (10^6^) were lysed (4 °C; 30 min) in 1X RIPA buffer (Cell Signaling Technology Inc., Danvers, MA, USA) containing protease inhibitors (PMSF, Thermo Fisher Scientific) and phosphatase inhibitors (PhosSTOP, Roche, Basel, Switzerland). Protein standards were calculated using the Bradford Assay, CBTM Protein Assay (G-Biosciences/Geno Technology, St. Louis, MO, USA). All samples were denatured (100 °C; 10 min) followed by SDS-PAGE and transferred to PVDF membranes. α/β-tubulin or β-actin were assessed as loading controls, and antibodies are listed in Supplemental Table S5.

### 4.6. Apoptosis Assays of FLT3+ AML Cells Expressing shPSMD3

MOLM-13, MOLM-14, and MV4-11 cells expressing shPSMD3 were treated with doxycycline (100 ng/mL, 72 h) with and without the indicated inhibitors. Apoptosis was measured by staining of resulting cells with both APC-conjugated AnnexinV (BioLegend Inc., San Diego, CA, USA) and 7-aminoactinomycin D (7-AAD, eBioscience at Thermo Fisher Scientific), and analyzed on a BD FACSCanto II (BD Biosciences, San Jose, CA, USA) flow cytometer. All flow cytometry data were analyzed using FlowJo (Ashland, OR, USA), and only late apoptotic events are reflected in the bar graphs.

### 4.7. Colony Formation Assays on FLT3+ AML Cells Expressing shPSMD3

Colony formation assays were used to evaluate the effects of shPSMD3 on MOLM-13, MOLM-14, and MV4-11 cell survival. Briefly, 10^3^ cells/dish were plated in 0.9% MethoCult (StemCell Technologies, Vancouver, BC, Canada) methylcellulose reagent as previously described [17]. AML cell lines were plated in cytokine-free conditions ± quizartinib (20 nM) to inhibit FLT and/or doxycycline (100 ng/mL) to induce the knockdown. The resulting colonies were then scored and quantified after 14 days of culture in a humidified chamber at 37 °C with 5% CO_2_.

### 4.8. NOD-Scid Il2rgamma^null^ (NSG) AML Xenografts

Using either shPSMD3 or the shNT control, MOLM-13 cells were stably transduced and then placed in puromycin (2 μg/mL, 72 h) for selection. Resulting cells (3 × 10^6^ cells/mouse, *n* = 4–5 mice/group) were then intravenously injected into sublethally irradiated (1 × 200 Rad, RS 2000 X-ray Biological Irradiator, Rad Source Technologies) NSG recipient mice (Jackson Laboratories, Bar Harbor, ME, USA) to produce AML cell line xenografts. To induce in vivo gene expression of the shPSDM3 or shNT vectors, recipient mice were placed on 625 mg/kg doxycycline hyclate chow (Envigo Teklad, Indianapolis, IN, USA #TD.01306). Recipient mice were inspected daily for visual signs of leukemia, and moribund mice were humanely euthanized. All experiments were performed with approval by the Texas Tech University Health Sciences Center at El Paso Institutional Animal Care and Use Committee.

### 4.9. NF-κB Luciferase Reporter Assays

To measure endogenous NF-κB transcriptional activity, AML cell lines expressing shPSMD3 or the shNT control vector were lentivirally transduced using a pGreenFire1-NF-κB Lentivector reporter system per the manufacturer’s instructions (System Biosciences LLC, Palo Alto, CA, USA; Supplemental Table S3). Cells at 50–70% confluency were transduced using the TransDux solution with media to a 1X final concentration. Stably transduced cells were selected for 10 days in the presence of Geneticin^TM^ (Life Technologies) and resulting cells expressing the pGreenFire1-NF-κB reporter system were seeded at 5000 cells per well using a sterile flat-bottom 96-well plate (Greiner Bio-One, Kremsmunster, Austria) in the presence of the indicated inhibitors. After 72h, luciferase reporter activity was analyzed using the ONE-Glo Luciferase Assay System (Promega Corporation, Madison, WI, USA) on a CLARIOstar Plus (BMG Labtech, Ortenberg, Germany) plate reader.

### 4.10. Co-Expression Network of PSMD3 and Functional Enrichment Analysis

The co-expression network of *PSMD3* in AML TCGA data was generated in UALCAN (http://ualcan/path.uab.edu/, accessed on 30 July 2021) and presented as a heatmap [59]. Pathway enrichment analysis of the co-expressed genes was performed using Metascape (https://metascape.org, accessed on 20 December 2021) [62].

### 4.11. Proteomics Sample Preparation and Analyses

Proteomics analyses were performed at the University of Texas Southwestern (UTSW) Medical Center Proteomics Core Facility (Dallas, TX, USA). Briefly, 10^6^ MOLM-13 and MOLM-14 cells and derivative lines were lysed in 1X RIPA buffer containing protease and phosphate inhibitors (Thermo Fisher Scientific). Samples were digested overnight with trypsin (Pierce^TM^, Thermo Fisher Scientific) after reduction and alkylation with dithiothreitol (DTT) and iodoacetamide (Sigma–Aldrich, Darmstadt, Germany). Samples then underwent solid-phase extraction cleanup with an Oasis HLB plate (Waters Corporation, Milford, MA, USA), and the resulting samples were injected onto an Orbitrap Fusion Lumos mass spectrometer (MS), Q Exactive^TM^ HF (Thermo Fisher Scientific) coupled to an Ultimate 3000 RSLC-Nano liquid chromatography system (Thermo Fisher Scientific). Samples were injected onto a 75 μm, 75-cm long EASY-Spray column (Thermo Fisher Scientific) and eluted with a gradient from 0–28% buffer B over 90 min. Buffer A contained 2% (*v/v*) acetonitrile (ACN) and 0.1% formic acid in water, and buffer B contained 80% (*v/v*) ACN, 10% (*v/v*) trifluoroethanol, and 0.1% formic acid in water. A positive ion mode was used with a source voltage of 1.8 kV, with an ion transfer tube temperature of 275 °C.

MS scans were acquired at 120,000 resolution in the Orbitrap (Thermo Fisher Scientific). Up to 10 MS/MS spectra were obtained in the ion trap for each full spectrum acquired, using higher-energy collisional dissociation (HCD) for ions with charges 2–7. Dynamic exclusion was set for 25 s after an ion was selected for fragmentation. The raw MS data files were analyzed using Proteome Discoverer v2.4 SP1 (Thermo Fisher Scientific), with peptide identification performed using Sequest HT searching against the human protein database from UniProt, which is a freely accessible resource for protein sequence and functional information. Fragment and precursor tolerances of 10 ppm and 0.6 Da were specified, and up to three missed cleavages were allowed. Carbamidomethylation of cysteine (Cys) was set as a fixed modification, with oxidation of methionine (Met) set as a variable modification. Pathway enrichment analysis of the dysregulated proteins was performed using Metascape [62].

### 4.12. Genomic Alterations of PSMD Subunits in AML

cBioPortal (https://www.cbioportal.org/, accessed on 30 July 2021) is an online cancer genomics database that was used for the analysis of genomic variations across various cancer types [63]. The database was used to analyze data sets that contained point mutations, deep deletions, and DNA amplifications for the different *PSMD* subunits that correlated with OS in AML, similar to our previous analysis performed in other types of cancers [11]. The data is represented as a bar graph for the various *PSMD* subunits in AML.

### 4.13. Statistical Analyses

All experiments were performed in triplicate unless otherwise noted. A two-tailed Student’s *t*-test was used to analyze cell line and mouse data demonstrating equivocal variance. Welch’s *t*-test was used to calculate significant differences in expression comparing normal versus AML specimens, or AML specimens based on clinicopathological features (wild-type versus mutated FLT3). For analysis of OS, primary AML samples were divided into high versus low PSMD subunit expression, with sample values higher than or lower than the 3rd quartile value, respectively, with data presented as Kaplan–Meier curves. For proteomics data analysis, each sample was normalized to its internal expression of GUS, and resulting values were analyzed with a Student’s *t*-test with a false-discovery rate (FDR) cutoff of 1% for all peptides. Error bars represent the standard error of the mean (SEM), and a *p*-value < 0.05 was considered statistically significant.

## Figures and Tables

**Table 1:** Non-ATPase subunits of the 19S proteasome complex.

Gene	Alias	Full Gene Name	Chromosome Location
*PSMD1*	*Rpn2*	Proteasome 26S Subunit, Non-ATPase 1	chr2:231,056,845–231,173,116
*PSMD2*	*Rpn1*	Proteasome 26S Subunit Ubiquitin Receptor, Non-ATPase 2	chr3:184,299,198–184,309,050
*PSMD3*	*Rpn3*	Proteasome 26S Subunit, Non-ATPase 3	chr17:39,980,807–39,997,959
*PSMD4*	*Rpn10*	Proteasome 26S Ubiquitin Receptor, Non-ATPase 4	chr1:151,254,709–151,267,479
*PSMD5*	*KIAA0072, S5B*	Proteasome 26S Subunit, Non-ATPase 5	chr9:120,815,496–120,842,951
*PSMD6*	*Rpn7*	Proteasome 26S Subunit, Non-ATPase 6	chr3: 64,010,550–64,024,010
*PSMD7*	*Rpn8*	Proteasome 26S Subunit, Non-ATPase 7	chr16:74,296,814–74,306,288
*PSMD8*	*Rpn12*	Proteasome 26S Subunit, Non-ATPase 8	chr19:38,374,550–38,383,824
*PSMD9*	*Rpn4*	Proteasome 26S Subunit, Non-ATPase 9	chr12:121,888,732–121,918,297
*PSMD10*	*Gankyrin, P28*	Proteasome 26S Subunit, Non-ATPase 10	chrX:108,084,207–108,091,549
*PSMD11*	*Rpn6*	Proteasome 26S Subunit, Non-ATPase 11	chr17:32,444,379–32,483,319
*PSMD12*	*Rpn5*	Proteasome 26S Subunit, Non-ATPase 12	chr17:67,337,916–67,366,605
*PSMD13*	*Rpn9*	Proteasome 26S Subunit, Non-ATPase 13	chr11:236,966–252,984
*PSMD14*	*Rpn11*	Proteasome 26S Subunit, Non-ATPase 14	chr2:161,308,425–161,411,717

## Data Availability

Publicly available datasets were analyzed in this study. These data are located at http://gepia2.cancer-pku.cn/, http://ualcan/path.uab.edu/, https://www.cbioportal.org/ and https://metascape.org.

## Supplementary Materials

The following supporting information can be downloaded at https://www.mdpi.com/article/10.3390/ijms232314586/s1.
