# Supplementary material for: 19S Proteasome Subunits as Oncogenes and Prognostic Biomarkers in FLT3-Mutated Acute Myeloid Leukemia (AML)
Source: Int J Mol Sci. 2022 Nov 23;23(23):14586. doi: 10.3390/ijms232314586 (PMC9740165; doi:10.3390/ijms232314586)
Supplement: Supplementary file 1 [file ijms-23-14586-s001.zip › ijms-1878742-supplementary.pdf]

SUPPLEMENTAL INFORMATION

19S Proteasome Subunits as Oncogenes and Prognostic Biomarkers in FLT3-Mutated Acute Myeloid Leukemia (AML)

Joshua J. Lara[1,2†], Alfonso E. Bencomo-Alvarez[3†], Mayra A. Gonzalez[3], Idaly M. Olivas[2,3], James E. Young[2], Jose L. Lopez[1], Vanessa V. Velazquez[1], Steven Glovier[2], Mehrshad Keivan[2], Andres J. Rubio[1-3], Sara K. Dang[1,2], Jonathan P. Solecki[2], Jesse C. Allen[1], Desiree N. Tapia[1], Boranai Tychhon[1], Gonzalo E. Astudillo[1], Connor Jordan[2], Darshan S. Chandrashekar[4], and Anna M. Eiring[1-3*]

[1]Paul L. Foster School of Medicine, Texas Tech University Health Sciences Center at El Paso, El Paso, TX, USA; [2]L. Frederick Francis Graduate School of Biomedical Sciences, Texas Tech University Health Sciences Center at El Paso, El Paso, TX, USA; [3]Center of Emphasis in Cancer, Department of Molecular and Translational Medicine, Texas Tech University Health Sciences Center at El Paso, El Paso, TX, USA; [4]Department of Pathology-Molecular & Cellular, University of Alabama at Birmingham, Birmingham, AL, USA.

*Corresponding Author: Anna M. Eiring, PhD; 5001 El Paso Drive, MSC 32002, MSB1 Room 2112, El Paso, TX 79905, USA; Ph: (915) 215-4812; E-Mail: anna.eiring@ttuhsc.edu.

† These authors contributed equally to this work.

SUPPLEMENTAL TABLES

Protein	Description	FC	Log2(FC)	TTEST
Proteins upregulated after PSMD3 knockdown				
NRM	Nurim	22.789	4.510	0.037
TAF6	Transcription initiation factor TFIID subunit 6	4.059	2.021	0.089
UBE3A	Ubiquitin-protein ligase E3A	2.596	1.376	0.074
RANBP9	Ran-binding protein 9	2.383	1.253	0.094
ATP6V0D1	V-type proton ATPase subunit	2.171	1.119	0.088
SNRPA1	U2 small nuclear ribonucleoprotein A	1.851	0.889	0.096
PDRG1	p53 and DNA damage-regulated protein 1	1.846	0.884	0.064
TMX4	Thioredoxin-related transmembrane protein 4	1.713	0.777	0.003
MLF2	Myeloid leukemia factor 2	1.696	0.762	0.079
MSH6	DNA mismatch repair protein Msh6	1.695	0.761	0.078
RPAP1	RNA polymerase II-associated protein 1	1.690	0.757	0.030
TMEM214	Transmembrane protein 214	1.650	0.722	0.012
RFC1	Replication factor C subunit 1	1.641	0.715	0.098
VPS36	Vacuolar protein-sorting-associated protein 36	1.630	0.704	0.007
PEBP1	Phosphatidylethanolamine-binding protein 1	1.627	0.702	0.061
NUDT1	7,8-dihydro-8-oxoguanine triphosphatase	1.625	0.701	0.031
CRYZ	Quinone oxidoreductase	1.563	0.645	0.049
MPI	Mannose-6-phosphate isomerase	1.552	0.634	0.039
Proteins downregulated after PSMD3 knockdown				
BLMH	Bleomycin hydrolase	0.690025	-0.53528	0.099088
EEF1A1	Elongation factor 1-alpha 1	0.661475	-0.59624	0.027017
RPL36	60S ribosomal protein L36	0.658609	-0.60251	0.026183
HBG1	Hemoglobin subunit gamma-1	0.645747	-0.63096	0.012411
MATK	Tyrosine-protein kinase	0.641135	-0.6413	0.054298
SYPL1	Synaptophysin-like protein 1	0.6184	-0.69339	0.072819
PRKAR2A	cAMP-dependent protein kinase type II-alpha regulatory subunit	0.55575	-0.84749	0.042249
PSMD4	26S proteasome non-ATPase regulatory subunit 4	0.472821	-1.08063	0.009376

Table S1. Up- and down-regulated proteins after PSMD3 knockdown in MOLM-13 cells.

Protein	Description	FC	Log2(FC)	TTEST
Proteins upregulated after PSMD3 knockdown				
LGALS9	Galectin-9	837.149	9.709	6.27E-05
DOCK10	Dedicator of cytokinesis protein 10	379.194	8.567	0.000285
LYZ	Lysozyme C	373.816	8.546	0.00301
KCTD12	BTB/POZ domain-containing protein KCTD12	250.127	7.967	0.000956
PSMB8	Proteasome subunit beta type-8	242.776	7.923	0.003202
PGM1	Phosphoglucomutase-1	219.534	7.778	0.000137
TRIM22	E3 ubiquitin-protein ligase TRIM22	179.361	7.487	0.010181
SLC16A3	Monocarboxylate transporter 4	149.597	7.225	0.000444
HLA-DRB1	HLA class II histocompatibility antigen, DRB1 beta chain	147.262	7.202	0.001186
ABRACL	Costars family protein ABRACL	141.745	7.147	2.39E-08
MNDA	Myeloid cell nuclear differentiation antigen	136.899	7.097	0.000189
IFI16	Gamma-interferon-inducible protein 16	114.283	6.836	6.49E-05
STK10	Serine/threonine-protein kinase 10	113.218	6.823	0.040061
SERPINB8	Serpin B8	102.974	6.686	0.044555
CRIP1	Cysteine-rich protein 1	102.304	6.677	0.006293
S100A4	Protein S100-A4	99.441	6.636	0.014281
MYO1G	Unconventional myosin-Ig	97.329	6.605	7.83E-05
ITPR1	Inositol 1,4,5-trisphosphate receptor type 1	83.020	6.375	0.015038
Proteins downregulated after PSMD3 knockdown				
PRAME	Melanoma antigen preferentially expressed in tumors	0.006	-7.280	0.000281
HBZ	Hemoglobin subunit zeta	0.010	-6.717	2.48E-06
SDF4	45 kDa calcium-binding protein	0.011	-6.555	0.000119
SLC38A2	Sodium-coupled neutral amino acid transporter 2	0.012	-6.440	0.000319
GATA1	Erythroid transcription factor	0.012	-6.343	0.000354
AKR1C2	Aldo-keto reductase family 1 member C2	0.013	-6.271	0.000372
NOP53	Ribosome biogenesis protein NOP53	0.014	-6.209	0.015708
DMD	Dystrophin	0.014	-6.191	0.02311
RHAG	Ammonium transporter Rh type A	0.015	-6.107	0.000107
UBP1	Upstream-binding protein 1	0.017	-5.911	0.011178
IFITM1	Interferon-induced transmembrane protein 1	0.017	-5.894	1.12E-05
LIN28B	Protein lin-28 homolog B	0.017	-5.854	9.44E-05
TMEM263	Transmembrane protein 263	0.018	-5.833	0.003264
VIPAS39	Spermatogenesis-defective protein 39 homolog	0.019	-5.692	1E-05
NFE2	Transcription factor NF-E2 45 kDa subunit	0.022	-5.506	0.005064
FADS3	Fatty acid desaturase 3	0.022	-5.479	6.66E-06
HBG1	Hemoglobin subunit gamma-1	0.023	-5.417	9.96E-05
CA1	Carbonic anhydrase 1	0.024	-5.382	2E-05

Table S2. Up- and down-regulated proteins after PSMD3 knockdown in MOLM-14 cells.

Vector	Vendor	Catalog #
psPAX2	Addgene	12260
VSV.G	Addgene	14888
shPSMD3	Cellecta, Inc.	CVSHC-PX
shPSMD3 Sequence: ACCGGCTGGGGTGTCTTGGTTTGTATGTTAATATTCATAGCATACAAGCCAAGACATCCCAGTTTT		
Non-Targeting shRNA (shNT)	Cellecta, Inc.	CVSHC-PX
pGreenFire1-NF-κB Lentivector	System Biosciences, LLC.	TR012PA-N

Table S3. Lentiviral vectors for virus production. NF-κB, nuclear factor-kappa B (p65); psPAX2, empty backbone 2nd generation lentiviral packaging plasmid; shRNA, small hairpin ribonucleic acid; shPSMD3, shRNA vector targeting proteasome 26S subunit, non-ATPase 3; VSV.G, envelope vesicular stomatitis virus G glycoprotein plasmid.

Name	Forward Primer (5'>3')	Reverse Primer (5'>3')
GUSB	GAAAATATGTGGTTGGAGAGCTCATT	CCGAGTGAAGATCCCCTTTTTA
PSMD3	ATCACGCCCGGGTCTATGAG	ATGCCGAAGCGTAGCTGTCC

Table S4. Primer sequences for RT-qPCR. GUSB, glucuronidase beta; PSMD3, proteasome 26S subunit, non-ATPase 3.

Name	Vendor	Catalog #
Rabbit polyclonal anti-α/β-tubulin	Cell Signaling Technology	2148S
Anti-rabbit IgG, HRP-linked Antibody	Cell Signaling Technology	7074
Rabbit polyclonal anti-PSMD3	Bethyl Laboratories Inc.	A303-826A
Rabbit polyclonal anti-Ub (FL-76)	Santa Cruz Biotechnology	sc-9133
Mouse monoclonal anti-β-actin	Sigma-Aldrich	A5441

Table S5. Antibodies for immunoblot. HRP, horseradish peroxidase; IgG, immunoglobulin G; NF-κB, nuclear factor-kappa B p65; PSMD3, proteasome 26S subunit, non-ATPase 3; STAT3, signal transducer and activator of transcription 3; Ub, ubiquitin.

SUPPLEMENTAL FIGURES

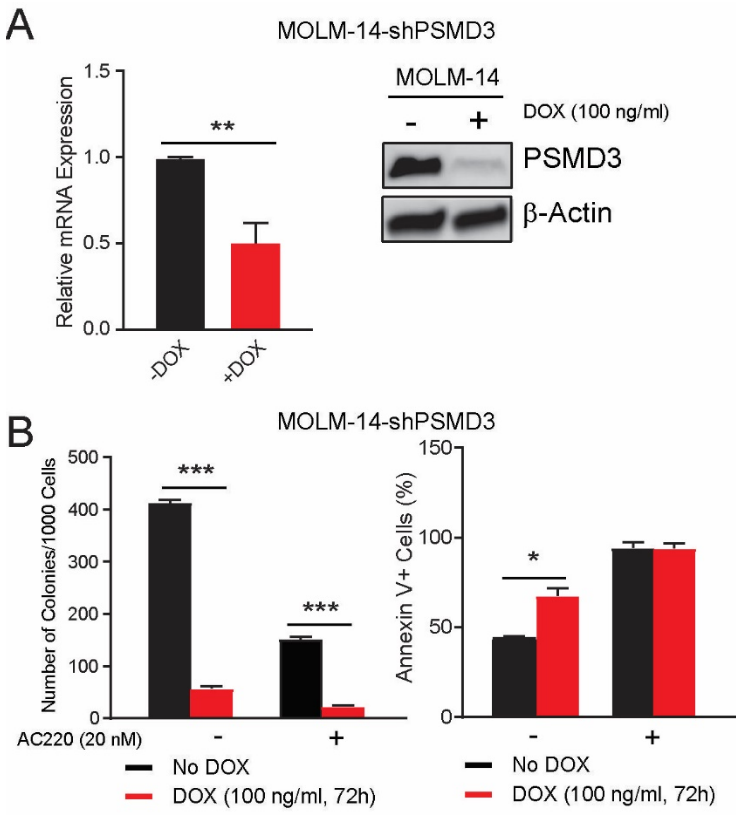

Figure S1. shRNA-mediated PSMD3 (shPSMD3) knockdown impaired survival of the FLT3-mutated AML cell line, MOLM-14, with significant effects on apoptosis. (**A**) Bar graph (**left**) and immunoblots (**right**) show PSMD3 knockdown at the mRNA and protein level, respectively, in MOLM-14-shPSMD3 cells cultured with and without doxycycline (DOX, 100 ng/ml, 72 h). (**B**) Bar graphs show the effect of shPSMD3 on survival in colony formation (**left**) and late apoptosis (**right**) in MOLM-14 cells cutltured ± doxycycline (DOX, 100 ng/ml, 72 h) and ± quizartinib (AC220, 20 nM, 72 h). Error bars represent the standard error of the mean (SEM). * $p < 0.05$; ** $p < 0.01$; *** $p < 0.001$.

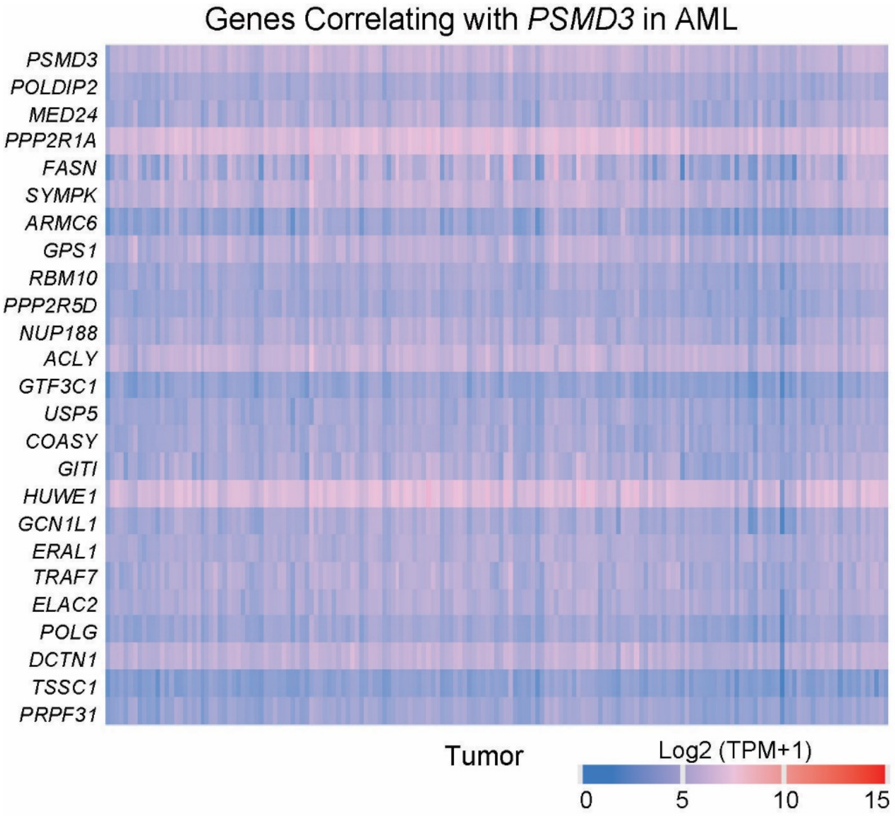

Figure S2. TCGA data available at UALCAN revealed the gene sets upregulated with *PSMD3* in AML. The heatmap shows the genes that are co-expressed with *PSMD3* in AML TCGA data, as calculated using UALCAN (http://ualcan/path.uab.edu/, accessed on July 30, 2021).

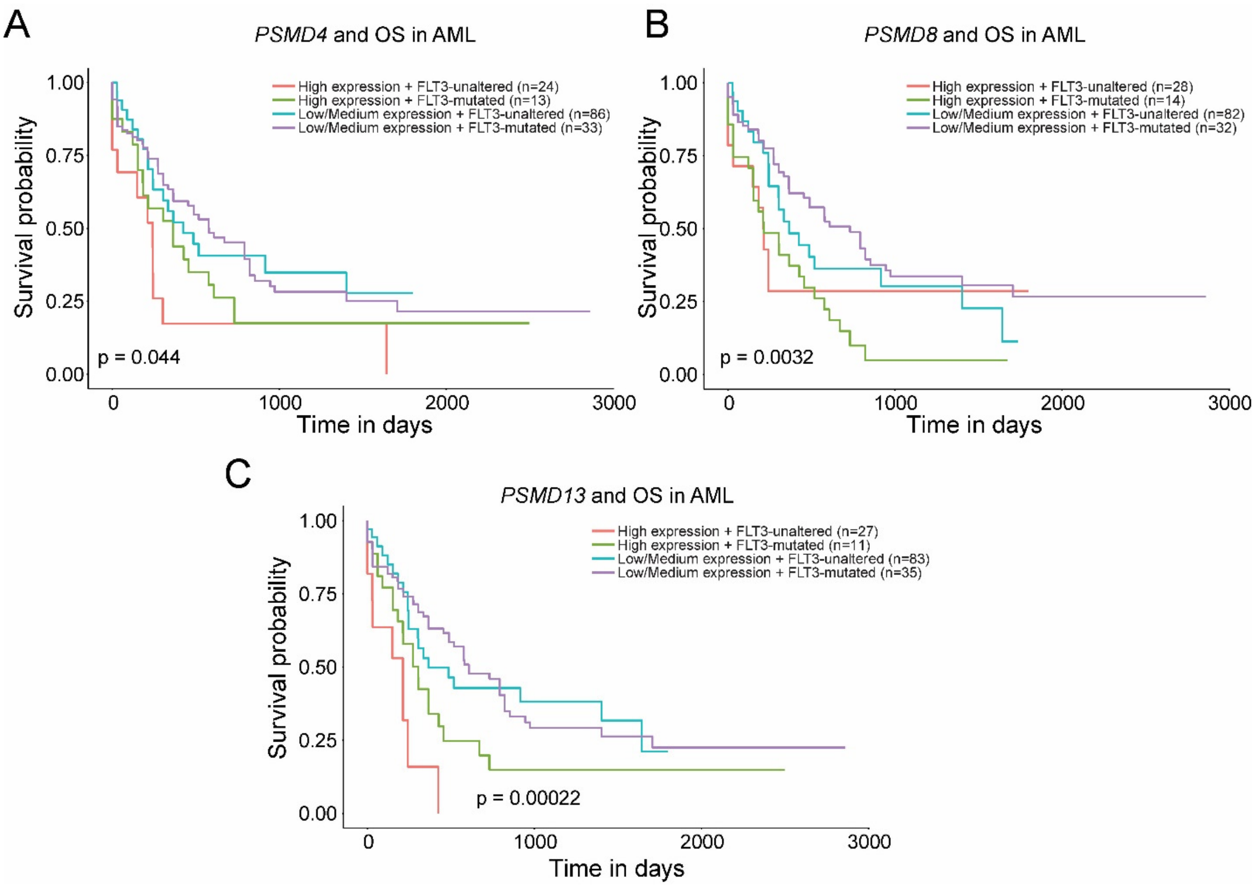

Figure S3. High levels of *PSMD4*, *PSMD8*, and *PSMD13* mRNA expression correlated with worse overall survival (OS) in acute myeloid leukemia (AML). (**A-C**) Kaplan-Meier curves generated in UALCAN (http://ualcan/path.uab.edu/, accessed on July 29, 2021) show OS for AML patients in the TCGA database, stratified by high versus low *PSMD4* (**A**), *PSMD8* (**B**), and *PSMD13* (**C**) mRNA expression levels and FLT3 mutation status.

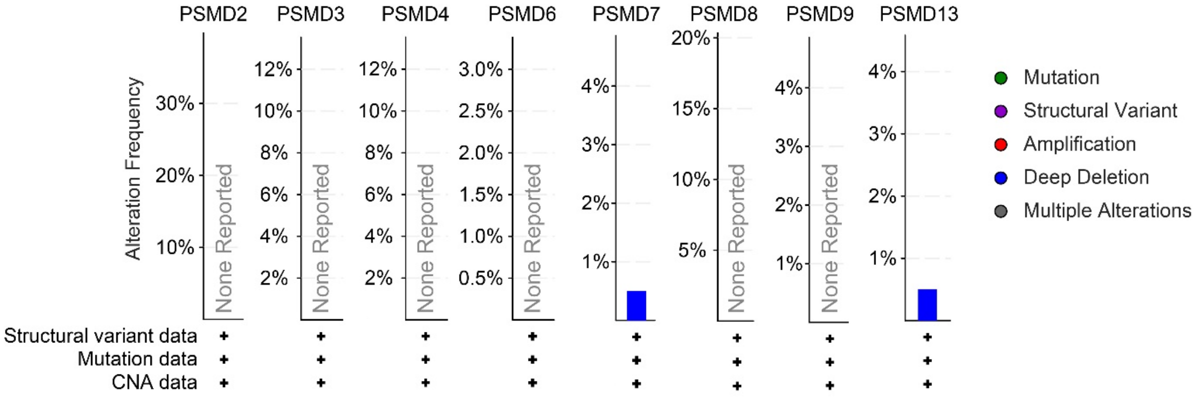

Figure S4. Genomic alterations in the genes encoding PSMD proteasome subunits whose expression correlated with OS in FLT3-mutated AML. Using data from cBioPortal (https://www.cbioportal.org/, accessed on July 30, 2021), we analyzed genomic alterations associated with *PSMD2*, *PSMD3*, *PSMD4*, *PSMD6*, *PSMD7*, *PSMD8*, *PSMD9*, and *PSMD13* in AML patients. Deep deletions were detected in the genes encoding *PSMD7* and *PSMD13*; no other genomic mutations were reported for these genes in AML.
